# Proceedings of Tri-State Dental Meeting, Held at Put-in-Bay, June 21, 22 and 28, 1898

**Published:** 1898-09

**Authors:** 


### PROCEEDINGS.

### Proceedings of Tri-State Dental Meeting, Held at Put-in-Bay, June 21, 22 and 23, 1898.

The meeting was called to order at 2:10 P. M., by Dr. Loeffler, of Michigan. It was opened with prayer by Dr. J. Taft.

The welcome address was delivered by Dr. Molyneaux, of Cincinnati, Ohio, President of the Ohio State Dental Society.

This was responded to, first by Dr. Loeffler, President of the Michigan Dental Association; also by Dr. Hartman, of the Indiana State Dental Association.

The first paper for the day was "Suggestion as a Factor in Controlling Patients During Dental Operations," by Dr. A. Jameson, Indianapolis, as follows:

A history of hypnotism, mesmerism, or animal magnetism, call it what you may, presents a series of theories and observations that are at wide variance, and are well calculated to fill the fair-minded man with distrust. But all through its history, though it may have been the instrument of charlatans and fakirs, there have been *results*, and these results were the cause of its investigation by scientific men, so that to-day it is no longer shrouded in mystery and darkness, but stands out a definite and tangible science, if you please, ready for our use if we choose to use it.

Hypnosis is of two degrees, the formal and informal. In the formal the hypnotist assumes complete mental control of the subject. In the informal there is a changeable degree of mental domination or influence. This latter state prevails usually without the knowledge of the subject, and ability to call it into play is one of the most useful qualities the dentist can possess.

We have all seen cases where an earnest, honest operator had but a meager clientele and an indifferent operator in the same community was always busy. The latter had the gift of producing informal hypnosis, or undue influence, while the former was devoid of it, and did not know what the trouble was.

The nerve cell and its attachment is the nerve unit and is called the "neuron." This neuron is composed of three parts, the cell, the axon and the tufted end of the axon. When we consider that there are 3,000,000 neurons in the body, nearly half of which are in the brain, and that each one, if not capable of contraction and expansion as a whole, at least has this power in its terminal filaments, we begin to realize what a delicate mechanism this is and how susceptible it must be to any force, or any impairment of its function.

Contact of these tufted axon ends is necessary for the transmission of impulse. Contact is produced by a lineal expansion of the tufted end. Contraction—making an imperfect contact of the tufts—prevails when the nerve is not employed in its physiological function. Relaxation or contraction of the various brain neurons constitutes sleep.

Hypnosis is an artificially induced relaxation of the neurons, in which state it is believed, any neuron or neurons may be impressed by "suggestion" on the part of the person who has induced this condition. The other neurons in the body are left relaxed.

Neurotic people, those with large, well-developed heads, full foreheads, large, dreamy eyes, high-arched brows, fine hair and soft skin, are easiest to hypnotize. They also make the best hypnotists.

We all use hypnotism to accomplish results. A great percent of dental operations are painless. With many patients the dread of the operation is the most worry, and after reaching the dental office informal hypnotism on the part of the operator—unconscious perhaps—enables the patient to undergo with ease operations that they had dreaded for weeks.

My idea in presenting this subject to a scientific body is that all things scientific are of interest to us when they play an important part in our life work. We are at all times searching for ideas and appliances that will make our work easier and better. This is characteristic of the dentist. But we are not taking up ideas that are recommended to us without reason. Hence, I have dwelt especially on the scientific side of this subject.

The discussion was opened by DR. C. B. BLACKMARR, Jackson, Mich.: When your committee asked me to open this discussion, I was told the subject was to be, "The Personal Element in Practice." When I received the printed program I was still sure of the subject, and felt as if I were ready for the same. Imagine my surprise when I heard the paper was on "Hypnotism." Dr. Jameson thought it would give you all a better chance to talk on this subject and down me. I have concluded not to be downed that way. Sidney Flowers, of Chicago, is an editor of a journal entitled, *Medical Hypnosis.* I will read you a few of the articles by Dr. Flowers. This is in the form of reports from different sources. First report is from Dr. Kingston, of the Kingston School of Therapeutics, in New York. He says: "Most people think hypnotism is a mysterious thing. People think we possess power to bring about cures very quickly. It depends upon the subject how long it will take to cure a disease. It is a subject of how soon the patient will lay aside all personal feelings and yield himself to operators. The operator, you will readily see, must have patience and perseverance.

"I had a patient who was very excitable, on entering my office. I asked her to be seated in the chair; on being seated she was very much excited, her teeth were badly decayed at that portion overlapped by the gums. My dental friend who had sent her to me had led her to believe that all she had to do was to visit me at once and she would have no pain. My treatment of this case was as follows: I impressed her with the fact that she must yield herself to me entirely. I placed her in the operating chair and told her to become perfectly relaxed and think of nothing but sleep, and close her eyes. (She would take these suggestions as I gave them). After a short while she said she was perfectly ready to believe and do what I said.

"I first impressed her with the fact that her teeth were as pieces of wood and she would feel no pain. After four or five of such treatments I could operate on her teeth and she would have no pain. The next day she came. I tried to impress her with the fact that she was created in a dentist's chair and that the dentist was going to work on teeth, but that she would have no pain during the operation. I took her to the dentist's chair for the operation, but as soon as the dentist commenced the work she had pain and would come out of her sleep. After this she related to me the great fear of her entire family of the dentist. I put her to sleep every day for more than a week, by the treatment as above given. Following this I gave her different treatment. I impressed her with the fact that her nerves were growing stronger and gums were becoming more healthy and when they were operated on they were not painful; and the gums did not feel like gums, but were free from sensibility. I ended by the suggestion that when she entered my office next time all sensibility of pain would leave her. I also gave her one suggestion —that she would have no pain when I ever touched her teeth, but on touching the dentine she had extreme pain. Further treatment consisted in impressing her with the fact that the operator on working on her teeth would not effect same. I impressed her with the fact that she would enjoy the whole operation, and when she awakened she would have no pain. She then went from my place to the office of the dentist and he worked on the teeth without any pain whatever, and she chatted with the doctor. She called on me the next morning and said she had no pain and never felt better in her life than now."

I will now give you my own method of treatment. It follows the above quite closely: "Every time I try to influence them I begin by a suggestion in reference to lessening pain, and follow it by suggestions already mentioned."

In general, I think that an anesthetic should be given in the office of a physician rather than the dentist, because if anything goes wrong the blame will be on the doctor. Some patients will not admit that the practitioner has better control than themselves.

Every good orator, in a sense, hypnotizes his audience. Every dentist can also, at the least, do it to the same extent. But the patient must be willing to be controlled by the dentist, because the dentist *must* control the patient. I would say to the therapeutic man, "The paths of pain are thine, go forth and relieve the suffering of this wrong."

This power will be a great boon to the dentist whose wife wishes a new bonnet, give her the suggestion she does not need it, and she will not want it. The same with the tramp, put him to sleep, suggest he likes work, and on awakening he will go to work. The Spanish need a few of such suggestions at this time.

The question was now open for general discussion and the presiding officer requested that each speaker kindly give his name and address, so the stenographer could get it.

Dr. Parker, of Grand Rapids: I think we have never had a better illustration of the power of suggestion than by the last two speakers. When Dr. Jameson sat down you were having a sense of the philosophic feeling about you. But when Dr. Blackmarr sat down you had the funny side of the question—he suggested it to you, and you saw it in that way. It has been a study of mine for a long time. My thought upon the matter is, if I were called upon to give a new definition of the "genus homo," it is that, "man is he who suggests." Every singer and every artist suggests, and the whole of life is suggestion. That it could be made to control pain it seems to me goes without saying. It certainly does to dentists who have thought of it along this line.

Dr. Meachen said: "If you suggest to a patient it is going to hurt, it will hurt. Take, for instance, the dental engine, which is a terror to most all. I simply say to the patient, it is a tool in my hands, if it hurts it is I doing it, and not the engine; it is just as harmless as the excavator. Of course, if you would get caught in a piece of machinery it would tear you to pieces, but this is not a machine but is an instrument in my hands; it will not hurt you any more than the excavator. I

will give an illustration of what shall be done to bring the patient to understand, along the line of suggestion: Some ten years ago I had for a patient a very bright lady, a teacher of drawing in our public schools. She had been practicing for quite a while what was then the fad, and known as mind-reading. She would do extraordinary things in this line. She came came to me for a dental operation, she had an exceedingly sensitive tooth, it was a lower molar. She said, 'Don't you think you can hypnotize me?' I said, I think I can if you will place yourself in such a position as I have seen in your seances. She said she could do it. I said, you go to sleep; I also said, you are down in a certain park in our town and you are in a hammock, and here is a book and it is an elegant story. I excavated the tooth and made the filling without any symptom of pain whatever. I said, 'all right.' She awakened and said she had been having a nice time; thought she was in the park, in a hammock, reading a delightful story. She said, 'What have you been doing all this time?' [Much laughter.] I never had another patient whom I thought I could control in that way."

Dr. W. C. Barrett, of Buffalo, N. Y.: Ever since the earliest times that hypnotism in some form has been used, there have been many wonderful things attributed to it. That these have all been reduced to a law I am not ready to accept. I do not believe in any of the stories that have been told by the advertising quacks, whether medical or dental, who have published articles and sent them out for advertising purposes. I am not one of the imaginative kind, it must take more than fictitious matter to impress my mind. It may be I have a peculiarly constructed mind, incapable of thinking of such things. A man in Chicago made prophecy: "That in five years every dentist who is worthy of practicing dentistry would be using hypnotism." I must be one of the unworthy. Down to the present time all the names which it has been called by have the same generic order, animal magnetism, second sight, spiritualism in many of its manifestations, clairvoyanc, mental telegraphy, etc., all belong to the same generic class. I know of many cases in which the practice of hypnotism has brought the most serious injuries to patients. I believe, and hope that the time will come, when this will be prohibited by law, except in persons peculiarly qualified for it, and I think if it should come to-morrow, the world would be better and dentistry would stand on a higher plane, and it would teach each one of us the use of the hypnotic power of kindness and delicacy of touch. We should not attempt by some kind of hypnotic power, by any kind of circus business whatever, to control it instead of by means which are natural and which every dentist should acquire.

Dr. G. E. Hunt, of Indianapolis: It is perhaps a little unfortunate that the word hypnotism was uttered here this afternoon at all. The word is so connected in our minds, with charlatans and quacks, and men who have some one to perform the antics for them, that it is a little difficult to disassociate our minds from that class of people. I do not know anything of hypnotism, I have seen people put to sleep, but whether a genuine case or not I do not know. Dr. Barrett says he does use hypnotism, but I believe he uses suggestion and that is the first step toward the hypnotic state. Dr. Barrett calls it kindness and that is a good name for it, but it is the same. If he impresses the idea that he is not going to hurt them he is using the first step of hypnotism. The hypnotists of today do not claim they have any supernatural power, or that they have any animal magnetism which passes to the patient. They claim that if the patient puts himself in a perfectly susceptible mood, he will obtund his own nervous sensitive end organs. They claim that by practice they have learned how to best perform the operation. The fact that it is dangerous for a man to practice hypnotism pure and simple is undoubtedly a dangerous thing to do and when the best hypnotists, that is the men who are scientific in the pursuit, will say it is dangerous for the novice. They may cause incalculable harm to the patient. No one can use it not having learned.

I do not believe you can destroy sensibility in any one without a chance of harm befalling them, but the chance is very much reduced and small indeed. I do not believe the essayist ever intended that you or I should ever entertain the practice of hypnotism. The first step should be cultivated. You do not need to call it hypnotism, only suggestion. You impress the patient that you are not going to hurt them and it will not be severe at all and you can do for them more than the man who has not got their confidence. That is a suggestion.

I attended a seance of hypnotism one day last week. It may all be a fake, but I don't believe a man could run needles through me if I could feel pain and were playing hypnotist. I do not think it is an especial advantage to patient. I do believe a little suggestion on the part of the operator goes a long way in relieving pain.

Dr. Taft suggested the discussion be limited to fifteen minutes. It was moved, seconded and carried to this effect.

Dr. Hartzell, of Minneapolis: I have had opportunity of noticing this wonderful force that we have which allows ourselves to control ourselves and only regret it has been termed hypnotism. Suggestion is the true name which we should incur in ourselves and patients. It is a recognized fact by the whole commercial world that suggestion is one of the most wonderful influences in doing the ordinary business of life. Notice the life of a child as soon as it is born. If the mother says, well Charlie says the child is bashful and does not make up with strangers, he will always be a bashful child. On the other hand if she says the child is not bashful and will make friends with everybody, the child will be accordingly.

The merchant recognizes it, walk down the street and you will find that they are tearing out all their old store fronts and are putting in large plate-glass windows and are displaying their wares to every passerby. Same with coffee vender by the aroma of the coffee you are enticed to partake.

I had the opportunity of observing the manipulation of one or two men who have disturbed the common atmosphere of state ethics in the past three years in the west. While entirely out of sympathy with these men in what they taught I am a firm believer that suggestion personally handled in the hands of the dentists is one of the most useful elements that we use in practice to control our patients. Not hypnotism, but suggestion.

Dr. Sweetnam, Manistee: I have practiced hynotism in dentistry. I have extracted whole sets of teeth with the use of it. I have hypnotized a patient to have a surgical operation performed at some other place. I do not practice hypnotism on account of the deep-seated prejudice against it which has not been reduced to a science. While I have noticed this state many times it was in ignorance, as I did not know what caused it, I know we can do these things without hurting the patient.

Dr. J. Taft, Cincinnati: I have nothing to add to this subject. You all know about as much about it as I. There is a very great difference of opinion of the various members of the profession in regard it. Those who have investigated it seem to like it; they do not know how it is accomplished, nor can they control it. In some cases it is all right but in others it fails. You all know, some persons have a power of influencing patients which is not possessed by others. It is merely a difference in the individual. Dr. Barrett would so control his patient as to render him comparatively painless. We all know that patients treated in the same way will exhibit different manifestations.

These experiments which are made and have been related seem to show beyond the ability of any one to favor the contrary, that there is absolutely by this process immunity from pain.

It is not sufficiently plausible to warrant its use as a general practice and even those who have gone into it must, as the late speaker intimated, become more and more conscious of all this opinion. It is also true that he should wish to attain the highest knowledge of all things which he practices. The fakirs and charlatans will use it as long as they make it payable. I do believe however, in the personal influence of one person over another, and I believe it is perfectly common and right for any one to influence his patient as far as he can, for his benefit, whether it is by sympathy or some unknown influence may be the difference of opinion among you all.

It ought to be the enquiry of every one, what can I do to investigate the pain which is connected with the teeth and mouth. What influence can I use, should be the study of all. I have very little sympathy with common hypnotism as an influence of general utility or general practibility.

DR. JAMESON: I do not wish to be misunderstood as to meaning of my paper, I did not mean hypnotism nor did I make any suggestion as to such.

---

SECOND PAPER—"DENTIST'S POSTURE AT OPERATING CHAIR."

BY ELIZA M. MOSHER, M.D., ANN ARBOR, MICH.

I am not a dentist; I would feel myself an interloper were it not for two reasons: One, several years ago it became my duty to extract a large number of teeth that brought me into your line a little, and the other reason is the importance of the subject which we are to consider at this hour—"Whether the position of the dentist over his chair is one which is harmful, or which in any way tends to injure his body or interfere with his symmetry." That we cannot decide until we have examined the body a few moments from the standpoint of mechanics. In order to take up the subject from this view, I shall use a model of the trunk which I made several years ago.

We have here the bones of the trunk, minus the arms, head and some ribes. The human skeleton consists of a pedestal, the pelvis, upon which is poised a long, slender, flexible column; to this and hanging forward from it are certain weights, the heaviest and largest extending forward and we call it the chest, made of ribs, cartilages and a sternum. Inside that circle are organs which increase the weight to the front and middle third of this long, flexible column. Then we have as other weights two arms hanging from it, connected from the scapula and shoulder blades. Weight two and three. Then we have again a heavier or lighter weight in the form of head, poised upon the top. Now, to bring that coarsely shaped structure into the upright and hold it there as it stands is rather a difficult matter, but when you remember that, in addition to all this, there are attached two columns, the legs, which, by muscles, can be swung out and placed underneath it, so that this disjointed structure is supported in the standing posture upon this foundation, or legs.

When we come to take all this into consideration, we realize that we have a very unstable body, built in an unstable way, and weighted in such a way as to lose its balance easily.

Now, by what force is it held in the upright? It is raised by means of muscles, which adjust these weights so that they balance each other and so placed that it can overcome force of gravity and hold itself into the upright position, being adjusted so as to balance each other from side to side and from forward to backward, the muscle force allowing it to drop back and rest.

It is the same in sitting as in standing, and from the time the little child raises its head for the first time from its mother's arm until the days of decrepitude and old age the work of muscles, or a large part of same, are used in adjusting and readjusting these weights so that the body is relieved from vital force to be used for something better.

The question arises, Is there any posture which we may call normal, one which is better than all others, one toward which the body should ever be turned? In order to find out whether or not there is, we immediately think of the two organs which are constantly at work, the heart and lungs.

We say if there is a normal posture it must be one which gives to these two organs, these tireless workers, the largest space and least friction. If such a posture can be found, and in addition, we find that all the other organs of the body are in their best place and are active, with the least friction and are filling up only their space we say, that position is the one which it was intended man should keep. If we come to study this structure we find that when the body rests firmly on the balls of the feet and lightly on the heels, when the pelvis is down in front and up on back carrying the gluteal weight high, in that position we find sternum and spine as far apart as is possible for them to be without muscular action. In this position heart and lungs have the best amount of space.

Our wonder was, I remember in my student days when I was studying the gluteal muscles, I could not understand why that great mass of muscles were piled up there; I saw no use for it. There is no more work to be done in that region than many others where the work is more important. It is now explained that while the muscle is there to do a certain amount of work in raising from the sitting posture, etc., there is no need of such a weight of muscle there for working contractions, but there is need for it to act as balancing power to the chest.

Commonly one arm balances another, but from before backward it is not symmetrical. It was a difficult problem for me to ascertain where the balancing weights were placed until one day I discovered it while studying a little statuette, that upon the position of the pelvis depends the depth of chest.

When a person stands his weight upon the heels and not on the balls of his feet, the chest is flattened and the gluteal region is lowered, and when the gluteal weight is high the sternum must go forward away from the spine, and we have lack of space for heart and lungs to do their work.

Every organ in the body is at its best position when in this normal position, the spine is in the upright and has normal curves and is in its best position of strength.

Now, a little further. Upon the position of the feet under the body, and the pedestal beneath same, depends the shape of the body above. I need only to unfasten these chains to prove my point. If I unfasten these chains and raise the pelvis only possibly an inch, you will see how immediately the spine has to change its shape from top to bottom. The spine changes shape, and, with cause, all the other parts of the trunk are changed in shape. The pedestal has been altered in its level, and until something is done for it, it cannot be in equilibrium. The spine must assume a shape which is natural. The muscles of the various organs of the body cannot work with the pelvis in this position. Now, again I will repeat the statement: Upon the position of the feet and pelvis depends the shape of the body. Upon these two things depends the ability of the body muscles of same to work at its good advantage. If you stop to think, you change your posture to suit the work you are doing. Part of the time we remove the weight on one foot and use the other as balancing weight. In working on the right foot we throw out the left to balance the body, and *vice versa.* Upon the position of feet and pelvis depends your ability to use your muscles to good advantage.

The body by remaining in certain postures continuously or using certain muscles repeatedly, the tissues on the one side become permanently short and the tissues which have been used long become lengthened and the inter-vertebral substance thins on one side and thickens on the other, so that the body attains a permanent shape into the position which it occupies most often, so that you and I, if our eyes are open to the subject, can, with perfect ease, tell in what position any individual occupied in his work; in other words, the trade mark of his work is built into his body. Whether harm comes from that is, to a certain extent, worth our while to consider. The body is constantly exposed to conditions which are not favorable to it (and yet it does not do its best work when it is interfered with) and built over into a shape which places its organs at a disadvantage.

The practical question which comes up this afternoon is this: The dentist, almost more than any other workman, (if I may call him so) is obliged to occupy a position over his patient which is far from the normal position of the body, that in which it can do its best work. In making this study, it has been very interesting to me to see the influence first on the spine. It is his good fortune that he is obliged to change from one foot to another, but he puts more weight on the left foot. It is his right hand which is steady and he must turn his face to the patient. Perhaps you do not know what it does to the back and various parts of the body. From a study of it upon a good many individuals, I find that the spine becomes permanently twisted outward and to the left, the ribs project backward on the right and recede on the left. At the upper part the twist continues at the top, so we have a rotation of the spine upon its own axis. Then you find that he has, in addition to this, a low left shoulder, which makes the two sides not symmetrical. The anterior wall of the body is shortened very materially. The space from the pelvis to top of the chest is shortened and flattened, flattened on the right and pushed out on the left. Now, in that position the harmful effect is the influence upon the lungs, especially upon the young man who is not extra strong. The indoor life and flattening of chest is, of course, a great wrong. Using muscles on one side and which are not developed on the other, puts the body at a disadvantage.

The profession of dentistry has now become so important and is so influential, it is bringing into it the best of our young men, and also ladies. It seems to me reasonable that we should consider this matter and see if there is not some way to prevent it. There are three suggestions which I would like to make, and which are worth following:

1st. Cannot dentists usually see if it is not possible to adopt some position, varying the position of the patient more than they do in order that they may vary their own position; so many young men lose their health in this work. I believe this can be done; arrange the patient a little lower in the horizontal or some other change which will enable you to occupy a position of body during all these hours of hard work which shall be better for the work of your bodies and shall give to your children greater strength than from parents who have been injured in this way.

2d. In the schools teach the students to use both hands equally. Dentists have done it. Our young men are not being systematically taught to use both hands in their work.

3rd. I recommend that our students in the schools should be required to take exercise every single day so as to overcome and remedy the harm which shall come from this peculiar posture. There is no reason why this should not be done. If every dentist would begin to take exercise once or twice a day for ten or fifteen minutes, which shall stretch out the short ligaments and shorten those which are getting long and filling out the body, a better state of health would be enjoyed. If the dentist would do this the condition would be obviated, even though he is obliged to work in very bad postures.

This was followed by a demonstration of exercises best adapted to overcome the evil influences of mal-position at the chair. It was conducted by Miss Alice G. Snyder, of The Ladies' Gymnasium, University of Michigan, Ann Arbor.

ORDER OF EXERCISES.

*Poising.*—1. Raise on balls of feet, stretch up with chest and head even with chin; 2, same as No. 1, but at same time raise arms shoulders high at sides and stretch out; 3, same as No. 1, but at same time raise arms front and up and stretch up.

*Leg.*—Take neck firm and keep in all; 1, flex leg; 2, flex thigh; 3, extend right foot to right, pointing toe down, same with the left; 4, extend right foot back, pointing toe down, same with the left; 5, arms out, shoulder high, palms up; (a) extend right foot back, toe down; (b) raise knee front and up; (c) point toe back; (d) replace; 6, neck firm, change diagonally forward to right, same with the left; 7, (a) raise up toes; (b) bend knees; (c) straighten knees; (d) lower heels, keep trunk erect on hips; at same time (a) raise arms shoulders high at sides; (b) raise overhead; (c) lower to shoulder, and (d) replace at sides.

*Head.*—1, bend backwards; 2, force chin back; 3, bend back, resisting with hands.

*Arms.*—1, (a) raise arms sideways and above head until fingers touch; (b) bring hands down behind head; (c) raise arms to vertical; (d) bring back and down; 2, (a) flex arms, fingers not touching the chest but pointing back and out; (b) extend arms up diagonally, palms front, fingers and elbows straight, and at the same time bend head backward and look up; (c) bring the head to position and arms to flex; (d) position, and 3, raise arms sideways, palms up, press back, with arms and head forward with chest.

*Trunk.*—1, neck firm, bend forward; 2, arms at shoulder high, palms up and bend forward; 3, raise arms front and up, bend forward; 4, (a) raise arms shoulder high at sides; (b) bend forward; (c) raise arms above head; (d) lower to shoulder high; (e) raise trunk; (f) position.

Opening discussion by Dr. T. A. Whitslar, Cleveland, Ohio.

I feel that the paper we have just heard has well repaid us for coming here. This is of great importance, and probably more so than how to put in an amalgam filling, or filling root canals. The subject has been so well written and discussed that I feel myself unable to add much to it. I will add only a few words in reference to the mechanism of the body, and that is about the spinal column and the head. Diff-rent than the lower animals the head of the human being is supported in the center by the spinal column. In the lower animals the head is supported at the back. Animals which walk upon all fours and whose heads are supported at the back are supported also by the ligamentum nuchæ at the back. Their head is not so large comparatively and is also supported by more muscles. The back or spinal column is different in man than in lower animals. When the spinal column was before us we saw it was not in a straight line but had four curves, and these are all for the special purpose given to us by the Lord to help support the thorax and organs within the same. This, with the aid of the muscles of the back, keeps the body in an upright position, and all these have to do with the maintenance of a good, healthy body. The value of any human being depends, of course, upon all functions of the body being in a perfect condition. Adding to this the great number of nerves, we have an idea of the mechanism of our bodies. We also have a loss of nerve tone and this is that from which we, as dentists, suffer.

The question is, is the dentist's occupation a healthful profession? There is an humble illustration which I read not long ago: Take a pitcher that is so constructed that it may be utilized to carry water or material in for a number of years, if it is only filled to a certain point, when filled beyond that it breaks and is no longer useful. The same way with our bodies in the practice of the profession. There is a limit to the endurance; the body is not built to withstand the great muscular strain which a man is exposed to in dentistry. There is many a man who can perform great tasks well, but whenever they go beyond that they become physical wrecks. The dentist who is capable in all operations is one who has his body in a healthful condition and whose nerve tone and digestion are correct. How are we to overcome the troubles which come to us as workers in this line? We have had exercises presented to us this afternoon which are of great assistance to us. If you are an observer, and I have tried to observe at this meeting the older men of the profession, particularly those who have used the old Archer chairs, they are the persons who have had to suffer from the ill effects of stooping over, whilst the younger men keep their shoulders level and remain fairly straight. To illustrate this point, I wish to speak one word. All modern chairs can be raised up or down, and we must use same so as to accommodate ourselves as a matter of health and protection. If we raise the back of chair and head-rest we put the patient's head in the normal position or a little more forward, and this we do sometimes while operating upon the lower teeth; but if you desire to operate upon the upper teeth, in order to bring the head back and throw the patient into a position for easy access, you must lower head-rest and thus throw the head back. We also have as a valuable adjunct the Watkins head-rest, which enables us to throw the head to one side or the other. Whilst we are operatng upon the lower teeth, especially into canals, we can gain a straight view by looking through the corner of mouth. We must take into consideration the line of axis of tooth. Now, the longitudinal axis of the molar teeth is in a line with the lower border of bone extending up to the opposite side of mouth, and if the operator, in working upon the left, stands on the right he would be in a direct line with the tooth, and if he would operate on the right side he would stand on the left. We also have the use of the mouth mirror and also stool. This latter, whilst I have never used it, I have observed by this demonstration this afternoon that the stool is a very healthful thing for us. A man stands on two legs, an animal upon four. The animal stands upon the toes. Man stands upon the foot, he is supported by only about one-half, but this is counter-balanced by ball of foot and toes. An instep is the mark of perfection of the human race. In London in years gone by when policemen were brought up for inspection their insteps were observed, and if they had a low one they were refused because it was said a man with a low instep could not walk as long as a man with a high one.

As a matter of especial interest I want to say this: Of the four billions of people upon the earth about one-fourth die under the age of six years and about one-half under the age of sixteen years. These figures are stupendous, but we who are in this work try to keep well and live to a good old age. To illustrate, I examined a number of back copies of *Cosmos*, over seven years, and picked out, at random, thirty-five deaths during this time and found that of these thirty-five dentists who had died, the average length of life was sixty-two years. That gives us a little satisfaction in knowing that while our profession is a little unhealthy, we live longer than do physicians and many other classes of people.

As to exercises I agree to say only a word. Those which we have had to-day are certainly very excellent. Prof. Anderson, of Yale, also gives many such exercises for us. He recommends the head resistance as a very excellent thing. The only disagreement I have with this class of exercise is, that it is too tame for any of us. We should get out with our bicycles. We have to learn what kind of exercise is most beneficial to us. Bicycle riding takes us out into the air.

One of the most beneficial exercises in which we can engage is that of fencing. This is the most excellent one for the dentist; as the body is not only supported upon a pedestal at hips, but also upon both legs, and he is able to carry himself in any direction, either forward or backward. I certainly want to thank Dr. Mosher for giving us such an excellent paper.

Dr. J. Taft: The matter of position in its relation to and influence upon the muscles of some parts of the body at least, is so apparent that hardly anything specially need be said upon this point. It should be borne in mind that interference is made by false position, indulged in so frequently by the dentist, upon the various organs and parts of the body. Pressure upon the digestive viscera necessarily interferes with their work. If the chest is contracted pressure is made upon the lungs and their work imperfectly performed, and respiration is defective. In the case of the dentist this is a very common fault.

By false position the lung cavity is much lessened and the organs not only impaired and injured permanently by this defect becoming a habit. In many instances this undue pressure is continued for a number of hours each day. Some operators stand at the chair from eight to ten hours a day, and much of that time in a wrong posture. Not only the lungs and respiration suffer in this way but the heart as well, its proper function should be as perfectly maintained as possible.

The viscera of the abdominal cavity undoutedly suffers in the same way, but possibly not to the same extent. The liver may also be injured by defective surroundings. In the last twenty-five years some improvement has been made in regard to the special posture of the dentist. The modes of procedure in dental work have so changed as to be decidedly to the advantage of the dentist. Perhaps the dental engine has brought about a more radical change in this respect than any other instrument or appliance; however, the mallet and the rubber dam have each accomplished much in this respect. Chairs have been greatly improved, so that it is now practicable to bring the patient into a position much more favorable for the operator than in former times.

But with all these there is much of faulty position indulged by the majority of operators, but may we not hope that with all improved appliances and awakened consideration of the subject, a great change for the better will in the near future be brought about?

A quarter of a century ago or more many of the best dentists in the country, those having large practice, involving great labor, broke down, and in a large degree, at least, on account of the conditions that have been referred to in this paper. There were many cases in which men's health and strength gave way, the condition resulting fatally; many others there were whose health was so impaired that, though they lived for many years, they were very much enfeebled and unable to practice as they once did.

The injury resulting in the way referred to not only pertains to the organs themselves, as for example the lungs, heart and digestive organs, but all parts of the body suffer because of failure of these vital organs. Nutrition impaired, all the body suffers, and the body, as a whole, necessarily suffers from imperfect elimination. Any interference with the action of the heart involves every organ and tissue of the body. The nervous system and brain have their full share of injury under such circumstances. Younger members of the profession who have entered it in recent years do not realize the embarrassment that was experienced by those who have gone before.

If this subject receives at the hands of the profession the consideration it justly deserves there will be a rapid and great improvement in the near future.

This subject should be discussed in our societies, schools and journals in such a way as to call attention generally to it.

Dr. George E. Hunt, Indianapolis: The suggestions of Dr. Mosher have presented themselves to all of us quite frequently in our lives while practicing. We may not have given the subject as much thought and reflection as Dr. Mosher, and consequently have not anticipated the bad results which may take place. We all have a warning before it occurs, we get tired and nervous. For this tired feeling I would like to offer three suggestions which I have practiced for a number of years. When I feel tired in my chest and had been in a cramped position, I take a wind instrument, the *bazoo*, for instance; or you can take the clarionet or brass instrument: in playing it I have to extend my lungs to their full extent and allow a very small amount of air to leave the lungs at a time; within ten minutes after practicing thus I have had relief, so you see I have found a very easy way of correcting that tired feeling and avoiding deformity. Dr. Anderson says that rapid breathing will also relieve it.

Another thing—getting tired in the neck—I cure this, or at least obtain relief from it. For a great many years I slept in bed without a pillow. It forces my head back, it relieves my bad feeling in the neck and I don't become stoop-shouldered.

Another thing is to relieve the muscles of the arm and chest. While I rest in bed I like to put one arm under my head and change about. These are a few simple means which I employ, and they have been very beneficial to me, and will perhaps prevent spinal curvature, weak lungs, etc. I offer these suggestions for all.

Dr. Clayton, of Indiana: There has been much said here about the posture of the body at the chair. I defy anybody to stand straight with the heels which are made on the fashionable shoes to-day. Although the exercises we have seen to-day are very good, yet they are too mild. If every dentist here spends one dollar for the U. S. Tactics and follows out instructions as therein given he will develop into as good a man as is found in the army, which the foreign armies and nations say is the most elegant army in the world. I have had some experience in the army, and would be there to-day if not too old, but if I could get there to-day I would.

If you will take these exercises, known as Tactics of American Volunteers, they will do you as much good as those seen to-day, only they are much more violent. There is one very excellent one for strengthening the muscles of back and shoulders—throw arms out and then back; also spinal motion.

Bicycles have also been mentioned as a means of exercise. It is all right if properly done, but I do not believe in these so-called *scorchers*. Man can not ride on all fours and be healthful. I want to see a man stand or sit upright, as God intended he should. If the scorcher wants to ride let him go, and the sooner he scorches himself out the better.

Dr. Mosher, closing remarks: I am very much obliged to you for all the points you have given me. As I got up to speak to this audience of fine looking men I said to myself, "I am afraid I am in the wrong place." Then I remembered those students at Ann Arbor, whom I had been watching, and over whom I am groaning. My plea is for the men who are coming into the profession that they shall be trained correctly in this direction.

One word about exercises: Perhaps you did not observe—which I see you did not—that most of the exercises are corrective exercises. While the violent exercises are all right, it starts the blood, yet the exercises or posture which has produced these changes has been a very gentle, steady, even exercise with the mallet or something else (I do not know the instruments), they influence all the muscles to go wrong, and just as gentle exercises are quite as sufficient to correct them, it is not necessary to take these hard, violent exercises. I would not approve anybody ever taking such vigorous work after standing at the chair so long a time; we must take easy and slow exercises. I must still say that I believe the best exercises for overcoming the harm which is done is to give the same sort of work for exercise which is the opposite of the one used. The bicycle, ridden correctly, is an exceedingly good thing, but on the other hand it tends to hang the head just as the dentist's work tends to carry it forward. In passing into the operating room in Ann Arbor I looked down the line of chairs and I could not see a head. I said, "Where are the heads?" Immediately every head popped up. I claim that this should be overcome. We should do work just to strengthen the muscles which have been overtasked.

Dr. Taft: Mr. President, I move a vote of thanks be extended to Dr. Mosher and Miss Snyder for their kindness in presenting this subject to us.

The motion was seconded and unanimously carried.

First session of meeting adjourned at 6:10 P. M.

